# Artificial intelligence-based predictive hemodynamic monitoring in conjunction with goal-directed therapy reduces duration, frequency, and severity of intraoperative hypotension in major maxillofacial and otolaryngological surgery—a prospective randomized controlled pilot trial

**DOI:** 10.1186/s44158-025-00331-1

**Published:** 2025-12-22

**Authors:** Amir Ali Akbari, Christian Koch, Götz Schmidt, Daniel Schmermund, Christine Langer, Fabian Edinger, Sara Marie Denn, Melanie Markmann, Michael Sander, Marit Habicher

**Affiliations:** 1 Department of Anesthesiology, Intensive Care Medicine and Pain Therapy, University Hospital Giessen, Justus Liebig University of Giessen, Giessen, Germany; 2 Department of Oral and Maxillofacial Surgery, University Hospital Giessen, Justus Liebig University of Giessen, Giessen, Germany; 3 Department of Otorhinolaryngology, Head and Neck Surgery, University Hospital Giessen, Justus Liebig University of Giessen, Giessen, Germany

**Keywords:** Intraoperative hypotension, Hypotension prediction index, Goal directed therapy, Acute kidney injury, Major maxillofacial–otolaryngological surgery

## Abstract

**Background:**

Intraoperative hypotension (IOH) during non-cardiac surgery is associated with increased risk of postoperative complications, including acute kidney injury, myocardial injury, stroke, and mortality. Artificial intelligence-based predictive hemodynamic monitoring using the Hypotension Prediction Index (HPI), combined with goal-directed therapy (GDT), has been proposed to reduce IOH. However, its effectiveness in major maxillofacial and otolaryngologic surgery remains unclear.

The purpose of the study was to assess whether HPI-guided management or classical GDT reduces IOH compared to standard care in patients undergoing major maxillofacial and otolaryngologic surgery.

**Methods:**

In this randomized controlled pilot trial at a university hospital, 75 patients were allocated to one of three groups: control (*n* = 25), HPI-guided GDT (*n* = 25), or classical GDT without HPI (*n* = 25). In the control group, the advanced hemodynamic monitoring was blinded to the anesthesiologist. IOH was defined as mean arterial pressure (MAP) < 65 mmHg for > 1 min. Primary endpoints were the number and total duration of IOH episodes. Secondary endpoints included the time-weighted average MAP < 65 mmHg (TWA65) and postoperative complications.

**Results:**

Seventy-four patients were analyzed. The HPI group showed significantly fewer IOH episodes (median 3.0 vs. 7.0; *p* = 0.02) and shorter IOH duration (7.0 min vs. 46.0 min; *p* < 0.01) compared to control. No significant difference was observed between the classical GDT and control groups. Secondary outcomes were comparable across all groups.

**Conclusions:**

HPI-guided hemodynamic management significantly reduces the frequency and duration of IOH in major head and neck surgery. Larger studies are needed to evaluate effects on clinical outcomes.

**Trial registration:**

The trial was registered on clinicaltrials.gov (NCT04151264) on 14th October 2019.

## Key points

- HPI guided hemodynamic treatment reduces the incidence and severity of intraoperative hypotension in patients undergoing major maxillofacial and otolaryngological surgery in comparison with standard care.
- GDT alone did not reduce the incidence and severity of intraoperative hypotension in patients undergoing major maxillofacial and otolaryngological surgery in comparison with standard care.
- HPI-guided hemodynamic treatment showed no significant impact on postoperative outcome.

## Introduction

Blood pressure monitoring and especially invasive blood pressure measurement is a cornerstone of anesthesia management. Intraoperative hypotension (IOH) during non-cardiac surgery is associated with an increased risk of acute kidney injury, myocardial injury, stroke, and mortality, and its prevention is therefore a key goal of intraoperative hemodynamic management [1–5]. Despite the lack of a universally accepted definition [6], recent consensus recommendations suggest maintaining intraoperative mean arterial pressure (MAP) above 60–65 mmHg and individualizing treatment according to the underlying cause of hypotension [7].

Intraoperative goal-directed therapy (GDT) aims to optimize patient-specific cardiovascular function by guided fluid administration and vasoactive support. Several systematic reviews and meta-analyses report that GDT can reduce complications and length of stay in selected surgical populations, although its overall effect on postoperative outcomes and its role in routine practice remain debated [8, 9]. More recently, large multicenter trials in major abdominal and gastrointestinal surgery have questioned whether classical cardiac output–guided GDT consistently improves clinical outcomes compared with standard care [10, 11].

Machine learning–based predictive monitoring may complement conventional GDT by allowing earlier recognition and treatment of impending hypotension. The Hypotension Prediction Index (HPI; Edwards Lifesciences, Irvine, CA, USA) uses features derived from the arterial pressure waveform to estimate the likelihood of IOH (MAP < 65 mmHg) before it occurs [12, 13]. Several studies in non-cardiac surgery have shown that HPI-guided hemodynamic management can reduce the incidence and duration of IOH [14–16]. Nevertheless, its impact appears to depend on the applied treatment algorithm and has not been evaluated in all surgical settings [17, 18].

Patients undergoing major maxillofacial and otolaryngologic surgery frequently present with significant comorbidities and require long procedures with controlled hypotension and complex reconstructive techniques, including microvascular free flaps. In this context, careful balancing of fluid therapy and vasoactive support is crucial for both bleeding control and flap perfusion [19–21].

Therefore, the aim of this randomized controlled pilot trial was to assess whether HPI-guided management or classical GDT reduces the incidence and severity of IOH compared with standard care in patients undergoing major maxillofacial and otolaryngologic surgery.

## Materials and methods

### Study design

This study was an investigator-initiated prospective randomized controlled single-blinded trial conducted at the University Hospital of Giessen, Justus Liebig University, in Germany. Ethical approval was obtained from the institutional ethics committee (Justus Liebig University ethics committee, Chairperson Prof. Dr. H. Tillmanns) prior to the commencement of the study (Reference AZ170/18) on the 31 st of August 2018. The trial was registered on clinicaltrials.gov (NCT04151264). Written informed consent was obtained from all participants at least 1 day before surgery by one member of the study team. Subsequently, patients were randomly allocated in a 1:1:1 ratio to one of three study groups (control, GDT-only, HPI-guided GDT). Simple randomization was performed using identically folded slips of paper labeled with the three group assignments. All slips were placed in a single opaque envelope containing an equal number of allocations per group, and the envelope was shaken before each draw. For each enrolled patient, one slip was drawn immediately after informed consent by a member of the study team who was not involved in intraoperative anesthetic management. The allocation written on the slip determined group assignment and ensured concealment until the moment of randomization.

Given the exploratory nature of the study and the lack of comparable research at the time of its initiation in 2018, the sample size was set at 25 patients per group, resulting in a total enrollment of 75 participants.

In addition to the standard patient monitoring system, all patients were monitored using the Hemosphere® Monitor paired with AcumenIQ® Sensors or FloTrac® Sensors (both from Edwards Lifesciences, Irvine, CA, USA) to enhance data acquisition and ensure precise hemodynamic monitoring for the analysis. The Hypotension Prediction Index (HPI) served as a predictive tool designed to anticipate IOH up to 15 min before its occurrence, defined as MAP < 65 mmHg for at least one minute. The scale ranges from 0 to 100, indicating the likelihood of impending IOH and the remaining time before its onset [22]. This machine learning-based algorithm utilizes features extracted from the pressure waveform and was initially derived from a large retrospective dataset of surgical patients [12].

All Patients received arterial line placement before the induction of anesthesia. The induction of general anesthesia was achieved using Fentanyl (1–3 µg/kg) or Sufentanil (0.2–0.4 µg/kg), Propofol (1–2 mg/kg), and Cisatracurium (0.15 mg/kg). Anesthesia maintenance was routinely carried out with sevoflurane at a minimal alveolar concentration (MAC) of 0.7–0.9, supplemented by a continuous infusion of Sufentanil (0.5–1 µg/kg/h) and/or Propofol, depending on the attending anesthesiologist’s clinical judgment. The depth of anesthesia was monitored and adjusted to maintain a bispectral index (BIS) score between 40 and 60, ensuring an appropriate level of anesthesia throughout the procedure. Up until the start of the surgical incision, no more than 500 ml of crystalloid fluids were administered; then, a fixed infusion rate of crystalloid fluid (Sterofundin®, B. Braun, Melsungen, Germany) at 4 ml/kg ABW/h was implemented during the intervention period.

### Management of the control group

In the control group, the Hemosphere® monitor was connected to the arterial line sensor but remained fully covered, and the alarms were silenced. The electronic signal from this dual-output arterial line sensor (Acumen IQ® or FloTrac®) was also integrated into the standard operating room monitoring system (IntelliVue®, Philips Healthcare, Best, Netherlands), equal with all other study groups. This provided real-time data on arterial waveform, MAP, systolic and diastolic pressure, and pulse pressure variation, as part of standard intraoperative care. Anesthesiologists thus treated patients based on hemodynamic variables displayed on the standard monitor. No specific protocol was employed in the control group, but the target for blood pressure monitoring was to maintain a MAP above 65 mmHg.

### Management of the GDT group

**Fig. 1:**
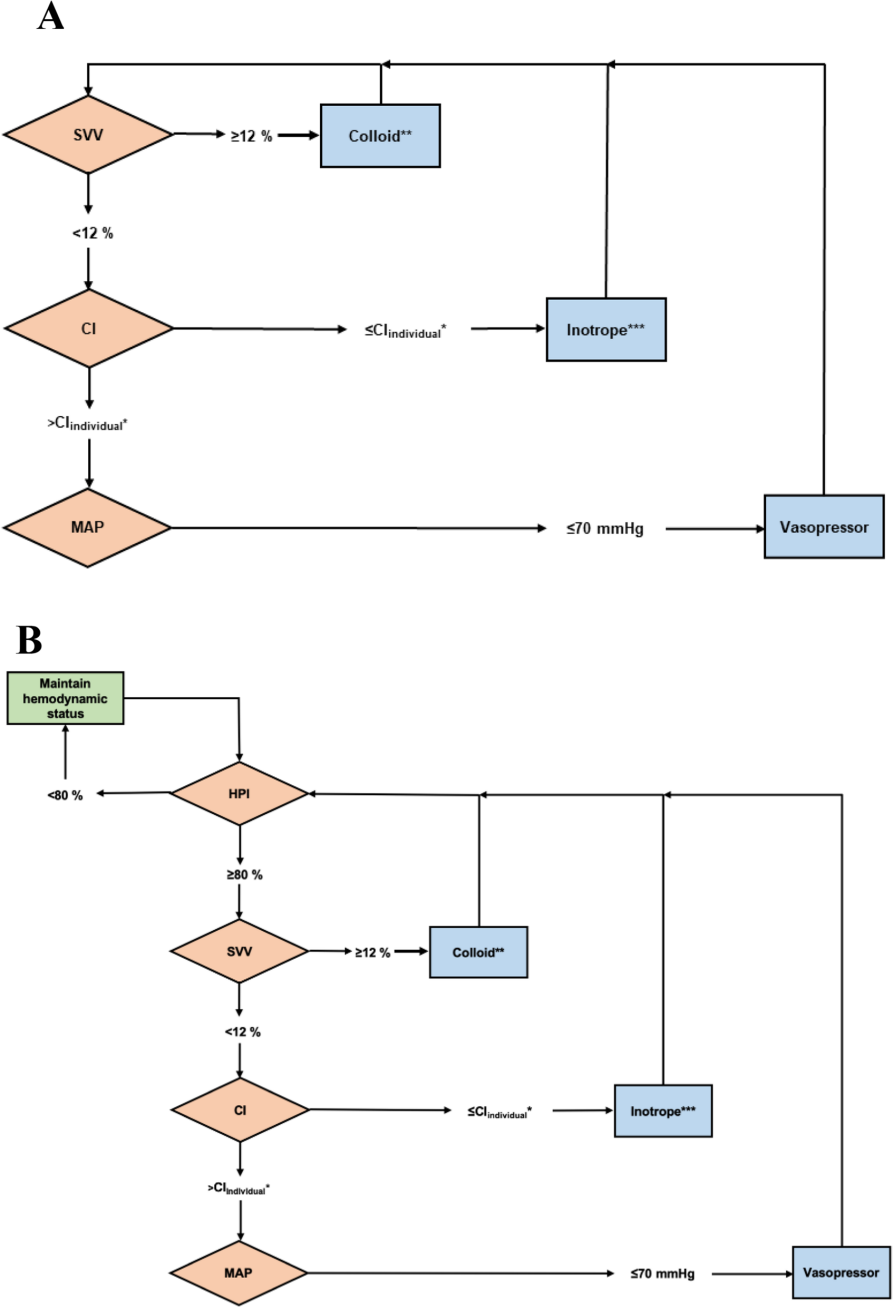
Treatment protocols. A. GDT-only protocol B. HPI-guided–GDT protocol

Additional to the standard monitor, the Hemosphere® monitor displayed extended hemodynamic variables derived from the arterial waveform every 20 s, and a differential treatment algorithm was implemented (Fig. 1A). For each patient, the individual awake resting cardiac index was determined prior to the induction of anesthesia.

The algorithm included the following steps:

1. If the SVV was considered above 12% under an adequate tidal volume (8 ml/kg IBW), a fluid bolus of 250 ml gelatine-based colloid (Gelafundin®, B. Braun, Melsungen, Germany) was administered. In cases where the patient exhibited an irregular cardiac rhythm, stroke volume variation (SVV) could not be reliably used. Instead, a target stroke volume (SV) was determined using an algorithm described in the “HIP-HOP” study published earlier by our group [23].
2. If the cardiac index remained below the identified individual cardiac index, inotropic therapy was initiated with dobutamine at an initial dose of 2.5 µg/kg/min. In case of a patient’s baseline CI below 2.0 l/min/m^2^, dobutamine was indicated between the beginning and CI increased above that cutoff.
3. If the heart rate exceeded 100 bpm for more than 20 min, a half dose of dobutamine was administered; if tachycardia persisted, the dobutamine infusion was discontinued.
4. If the cardiac index exceeded the defined individual cardiac index, or if it was treated with dobutamine and MAP remained equal to or less than 70 mmHg, vasopressor therapy was initiated.

### Management of the HPI group

In this group, the arterial line was connected to an AcumenIQ® Sensor which additionally enabled the HPI. The individual awake resting cardiac index was also defined for each patient before the induction of anesthesia. During surgery, if the HPI increased to 80% or more, the responsible anesthesiologist followed the same hemodynamic algorithm (Fig. 1B) to diagnose and treat the underlying problem leading to the risk of impending hypotension.

### Primary and secondary endpoints

The primary outcome of the study was the absolute number and duration of intraoperative hypotension, defined as a MAP below 65 mmHg for at least one minute during the incision–suture time. Thus, short hypotensive events (< 1 min) were not included.

Secondary endpoints included relative (i.e., in relation to the observational period/incision-to-suture time) number and duration of hypotensive events. Additionally, the area under the MAP 65 mmHg (AUC65) threshold and the time-weighted average (TWA) of MAP < 65 mmHg were calculated to compare our results to other studies. The TWA combines the duration and severity of hypotension, adjusted for the total time of observation. This metric is determined by calculating the AUC65 divided by the total duration of observation. The AUC65 is computed by multiplying the mmHg under the cutoff by the minutes, as described elsewhere [16, 24].

Postoperative outcomes included screening for acute-kidney-injury (AKI, measured by an increase of serum creatinine > 1.5 × above baseline or an increase of 0.3 mg/dl in 48 h according to the KDIGO definition) [25], a new-onset atrial fibrillation, the incidence of postoperative complications (overall, pulmonary, cardiac, infectious, and bleeding), intensive care unit (ICU)–length of stay, and hospital length of stay.

### Data collection

Intraoperative data were extracted from the Hemosphere® Monitoring platform as MS Excel® files, resulting in hemodynamic data with a resolution of 20 s. Additional intraoperative data were extracted from the on-site anesthetic data management and documentation system (NarkoData®, IMESO, Giessen, Germany). Furthermore, an adjunct paper-based case report form was filed where basic characteristics, interventions, and outcomes were documented.

### Protocol adherence

To quantify adherence to the study protocol, the electronic patient data management system was reviewed for all hypotensive events and corresponding interventions. Specifically, all interventions triggered by the HPI were assessed for both accurate indication and the appropriate therapeutic actions that followed in the HPI group. A ratio was then computed, representing the number of correct interventions divided by the total number of conducted, missing, or wrong HPI-triggered therapeutic actions. This ratio served as a measure of adherence to the study protocol in terms of responding appropriately to future hypotensive events identified by the HPI. In the GDT group, this was done separately in a similar way regarding the underlying algorithm.

### Statistical analysis

Given the absence of prior studies evaluating a reduction of hypotension through Hypotension Prediction Index (HPI)-guided monitoring in major maxillofacial and otolaryngological surgery, a sample size calculation was not performed. As this study is a pilot trial, we planned to include a total of 75 patients (25 per group) to assess early whether changes in hemodynamic measurement through the implementation of GDT, or GDT based on HPI, would result in a lower incidence and severity of IOH compared to standard management in this patient cohort. R (Version 4.3.2) was used for statistical analysis. The Shapiro–Wilk test was applied to test for normal distribution. For normally distributed values, one-way ANOVA with post-hoc Bonferroni tests was used to identify inter-group differences and test for statistical significance. The Kruskal–Wallis test in conjunction with post-hoc Nemenyi tests was used for non-normally distributed data, respectively.

## Results

Seventy-five patients were included in the study between September 2019 and July 2022. One patient in the control group was excluded due to technical reasons, resulting in missing data from the HemoSphere® monitoring system, preventing the accurate calculation of the AUC and TWA of hypotension. Therefore, 74 out of 75 patients were analyzed.

The baseline characteristics did not show any differences between the groups (Table 1).

**Table 1:** Baseline characteristics

Characteristic	Control group (*n* = 24)	GDT-only group (*n* = 25)	HPI group (*n* = 25)
Age (years)	69.2 ± 12.2	61.9 ± 10.1	66.4 ± 11.0
ASA class 2	11 (45.8)	13 (52.0)	6 (24.0)
ASA class 3	13 (54.2)	12 (48.0)	19 (76.0)
BMI (kg/m^2^)	25.7 ± 3.9	26.5 ± 5.1	25.4 ± 4.2
Male n (%)	16 (66.7)	15 (60.0)	14 (56.0)
Art. hypertension (%)	17 (70.8)	12 (48.0)	16 (64.0)
CAD (%)	5 (20.8)	3 (12.0)	3 (12.0)
COPD (%)	2 (8.3)	4 (16.0)	5 (20.0)
DM (%)	4 (16.7)	4 (16.0)	1 (4.0)
CHF (%)	2 (8.3)	2 (8.0)	1 (4.0)
CKD (%)	2 (8.3)	1 (4.0)	1 (4.0)
PAD (%)	2 (8.3)	2 (8.0)	4 (16.0)
AF (%)	3 (12.5)	0 (0.0)	3 (12.0)
Previous MI (%)	2 (8.3)	2 (8.0)	0 (0.0)
preOP Hb (mg/dl)	13.8 ± 1.7	14.0 ± 1.6	13.3 ± 1.6

Data are represented as mean ± standard deviation and number (%)

*AF* atrial fibrillation, *BMI *body mass index, *CAD *coronary artery disease, *CKD *chronic kidney disease, *COPD *chronic obstructive pulmonary disease, *DM *diabetes mellitus, *HI *chronic heart failure, *MI *myocardial infarction, *PAD *peripheral arterial disease

### Primary outcome

The number of hypotensive episodes was significantly lower in the HPI group compared to the control group (3.0 [0.0–5.0] vs. 7.0 [2.8–11.0], *p* = 0.02), and the absolute duration of hypotension was notably shorter in the HPI group (7.0 min [0.0–15.3] vs. 46.0 min [10.8–66.8], *p* < 0.01). However, there were no significant differences between the GDT group and the control group (Table 2).

**Table 2:** Hypotension: primary and secondary endpoints

Study group	IOH count (*n*)	Absolute IOH duration (min)	AUC (mmHg × min)	TWA (mmHg)
Control	7.0 [2.8–11.0]	46.0 [10.8–66.8]	167.33[34.3–323.9]	0.39 [0.14–0.91]
GDT-only	3.0 [1.0–8.0]	9.7 [2.3–30.7]	31.3 [9.3–136.0]	0.08 [0.03–0.31]
HPI	3.0 [0.0–5.0]	7.0 [0.0–15.3]	32.7 [0.0–79.3]	0.07 [0.00–0.29]
*p*-values				
Control vs. GDT	0.33	0.16	0.13	0.06
Control vs. HPI	**0.02**	** < 0.01**	**0.01**	**0.01**

Data are represented as median [25th–75th percentile]

Values shown in bold denote statistically significant differences between groups (*p*<0.05)

### Secondary outcomes

**Fig. 2:**
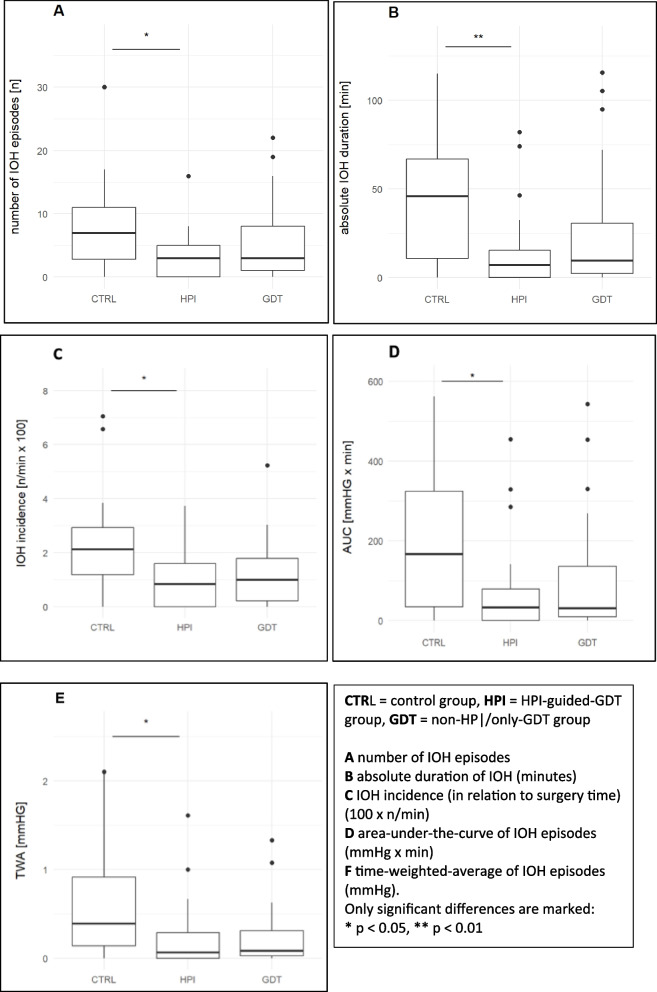
IOH-related primary and secondary outcomes

Additionally, both the AUC65 (32.7 mmHg × min [0.0–79.3] vs. 167.33 mmHg × min [34.3–323.9], *p* = 0.01) and the TWA of MAP < 65 mmHg (0.07 mmHg [0.00—0.29] vs. 0.39 mmHg [0.14–0.91], *p* = 0.01) were significantly lower in the HPI group compared to the control group. The GDT group did not show any significant differences regarding the hypotension-associated parameters compared to the control group. All data regarding hypotension are shown in (Table 2 and Fig. 2).

### Intraoperative hemodynamic and fluid data

Notably, intraoperatively both the HPI group and GDT group received significantly different amounts of colloids, with the GDT group receiving the most amount of colloidal fluids. Furthermore, the patients from the GDT and HPI group received significantly more inotropes, as it was a part of the hemodynamic algorithms.

Furthermore, patients in the control group had a significantly lower mean cardiac index during surgery compared to the GDT group. However, this difference was not observed between the control group and the HPI group. Interestingly, the mean heart rate in the control group was also significantly lower than in the GDT group throughout the operation, while no significant difference was found between the control group and the HPI group. All intraoperative data are shown in Table 3.

**Table 3:** Intraoperative data

Characteristic	Control group (*n* = 24)	GDT-group (*n* = 25)	HPI group (*n* = 25)	*p* CTRL vs. GDT/ CTRL vs. HPI
Anesthesia time (min)	461.5 [385.0–535.8]	488.0 [435.0–591.0]	461.0 [390.0–567.0]	0.38/0.94
Surgery time (min)	364.0 ± 145.8	401.4 ± 115.4	368.8 ± 110.7	0.89/1.00
Baseline cardiac index (L/min/m^2^)	2.9 [2.7–3.6]	2.8 [2.5–3.5]	2.5 [2.3–3.0]	0.91/0.11
Mean intra-operative CI (L/min/m^2^)	**2.5 [2.4–3.0]**	**3.1 [2.7–3.8]**	3.0[2.4–3.3]	**0.04**/0.39
Mean intra-operative MAP (mmHg)	75.2 ± 5.3	75.3 ± 3.5	78.7 ± 5.9	1.00/0.05
Mean intra-operative HR (min)	**63.4 [58.8–70.9]**	**75.8 [68.2–80.0]**	69.6 [59.2–77.6]	**0.02**/0.34
Cumulative dobutamine dose (mg)	**0.0 [0.0–0.0]**	**79.2 [37.0–111.0]**	**47.1 [30.0–90.0]**	** < 0.01/< 0.01**
Cumulative norepinephrine dose (µg)	509.0 [30.0–1125.3]	1220.0 [499.4–3251.8]	590.0 [0.0–2521.0]	0.09/0.60
Overall fluid input (mL)	3750.0 [3087.3–4661.8]	4875.0 [3670.0–5380.0]	4166.0 [2952.0–4954.0]	0.13/0.77
Crystalloids (ml)	2851.8 ± 1037.4	2698.4 ± 571.7	2581.3 ± 679.9	1.00/0.70
Colloids (ml)	**500.0** **[0.0–1000.0]**	**1250.0** **[1000.0–2000.0]**	**1000.0** **[750.0–1500.0]**	** < 0.01/0.02**
RBC (ml)	0.0 [0.0–375.0]	0.0 [0.0–600.0]	300.0 [0.0–600.0]	0.87/0.41
FFP (ml)	0.0 [0.0–0.0]	0.0 [0.0–500.0]	0.0 [0.0–500.0]	0.90/0.88
Overall fluid output	2199.4 ± 1251.0	2664.4 ± 957.2	2146.2 ± 962.4	0.39/1.00/
Blood loss (ml)	525.0 [387.5–825.0]	500.0 [400.0–800.0]	600.0 [300.0–1000.0]	0.90/0.94
Urinary output (ml)	1252.7 ± 761.8	1769.4 ± 715.3	1351.4 ± 798.8	0.06/1.00
Overall fluid balance (mL)	1725.3 ± 1021.6	2144.0 ± 1408.9	2161.9 ± 1390.4	0.78/0.72
PostOP Hb (mg/dl)	10.8 ± 1.6	10.6 ± 1.1	10.6 ± 1.5	1.00/1.00

Data are respresented as mean ± standard deviation and median [25th–75th percentile]

Values shown in bold denote statistically significant differences between groups (*p*<0.05)

The compliance to the protocol was 64.4% in the GDT group and 70.6% in the HPI group.

### Clinical outcomes

Clinical outcomes, such as length of ICU and hospital stay, and hospital mortality did not show any statistically significant differences between the groups (Table 4). The overall complication rate and the incidence of different complications do not differ between the groups. All postoperative outcomes are shown in Table 5.

**Table 4:** ICU and hospital stay

Characteristic	Control group	GDT-only group	HPI-group	*p* (CTRL vs. GDT/CTRL vs. HPI)
ICU stay (h)	22.3 [18.4–122.5]	22.0 [19.0–63.5]	20.0 [18.0–23.0]	1.00/0.31
Hospital stay (d)	14.0 [11.8–21.0]	14.0 [11.0–21.0]	15.0 [11.0–18.0]	1.00/0.90

**Table 5:** Postoperative complications

Complication	Control group	GDT-only group	HPI-group	*p* (CTRL vs. GDT/CTRL vs. HPI)
Overall *n* (%)	11 (45.8)	14 (56.0)	11 (44.0)	0.57/1.00
Pulmonary *n* (%)	8 (33.3)	6 (24.0)	4 (16.0)	0.54/0.20
Cardiac *n* (%)	2 (8.3)	3 (12.0)	0 (0.0)	1.00/0.23
Infectious *n* (%)	8 (33.3)	4 (16.0)	5 (20.0)	0.20/0.35
Bleeding *n* (%)	5 (20.8)	6 (24.0)	5 (20.0)	1.00/1.00
New onset atrial Fibrillation *n *(%)	1 (4.2)	0 (0.0)	0 (0.0)	0.49/0.49
AKI *n* (%)	2 (8.3)	1 (4.0)	0 (0.0)	0.96/0.87

## Discussion

In this randomized controlled pilot trial in major maxillofacial and otolaryngologic surgery, HPI-guided hemodynamic management significantly reduced the number and total duration of IOH episodes, as well as AUC65 and TWA MAP < 65 mmHg, compared with standard care. In contrast, a classical GDT protocol without HPI did not significantly differ from the control group. These findings suggest that a predictive, algorithm-based approach can improve intraoperative blood pressure stability in this complex surgical population.

Protocol adherence was moderate in both intervention groups but remained above 60% (70.6% in the HPI group and 64.4% in the GDT group). Despite this, HPI-guided management still achieved a clinically relevant reduction in hypotension, indicating that predictive triggers may facilitate earlier and more targeted interventions even when algorithms are not followed perfectly.

Patients in the GDT and HPI groups received more colloids and more inotropes than those in the control group, reflecting the protocolized nature of hemodynamic management, whereas the overall fluid balance was not different between the groups.

This observation suggests that fluid administration in the HPI group was more selectively targeted towards episodes of impending hemodynamic instability as indicated by a rising HPI value, rather than following a protocol aimed at proactively and continuously maximizing stroke volume as in the classical GDT approach. As a result, the HPI-guided algorithm may represent a more fluid-restrictive strategy, potentially avoiding unnecessary fluid loading and its associated risks. It is also worth noting that, although the difference did not reach statistical significance, the GDT group received the highest total volume of fluids among all groups.

Interestingly, the GDT group showed higher intraoperative cardiac index and heart rate than the control group, whereas these differences were not observed between the HPI and control groups. Notably, the absolute dosage of dobutamine used in the study was highest in the GDT group, although this difference was not statistically significant compared to the HPI group.

This pattern suggests that the HPI-guided algorithm targeted interventions more selectively in response to impending hypotension, rather than continuously maximizing cardiac index.

The routine approach of maximizing stroke volume and cardiac index, which has been a cornerstone of GDT over the past decade, has recently been shown to offer limited improvements in outcomes and may even lead to undesirable side effects such as fluid overload and cardiac complications. In the Optimise II trial, the primary endpoint of postoperative infections did not differ significantly between the intervention and control groups; however, patients in the intervention group experienced a significantly higher incidence of postoperative arrhythmias compared to the control group. This increase may be attributed to the use of inotropes, such as dobutamine or dopexamine, which were administered to maximize stroke volume in the intervention group [11].

Our results are consistent with previous studies reporting that HPI-guided hemodynamic management reduces IOH in non-cardiac surgery [14–16, 24]. At the same time, recent work has raised methodological questions regarding the validation of HPI and has suggested that, under some conditions, simple MAP-based thresholds might predict hypotension with comparable performance [26–28]. The present trial was not designed to resolve these methodological issues but provides complementary clinical data in a specific high-risk head and neck population, in whom HPI-guided management clearly reduced IOH relative to standard monitoring.

Despite the marked reduction in hypotension, we did not observe significant differences in postoperative complications, ICU or hospital length of stay, or mortality between the three groups. This is in line with larger trials in abdominal surgery, in which HPI-guided therapy did not reduce AKI or other major complications compared with standard care [18].

However, our study was not powered to detect differences in postoperative complications, which limits the conclusions that can be drawn regarding these outcomes.

Taken together, our findings indicate that HPI-guided management can reliably reduce the incidence and severity of IOH in major maxillofacial and otolaryngologic surgery, whereas a classical GDT protocol alone may be insufficient in this setting. Future research should clarify whether specific subgroups—such as patients with extensive free flap reconstruction, pre-existing cardiovascular disease, or very long operative times—derive particular clinical benefit from predictive hemodynamic monitoring and how simple, user-friendly treatment algorithms can best be integrated into routine perioperative care.

Although the results suggest that while HPI-guided therapy effectively predicts intraoperative hypotension, it does not necessarily translate into improved postoperative renal outcomes or reduced complications in various surgery patients. This highlights the need for further research to determine which surgical populations and conditions may benefit most from HPI-based management.

### Limitations

First, it was designed as a single-center pilot trial with a relatively small sample size and was not powered to detect differences in postoperative complications or mortality. Second, baseline cardiac index was measured immediately before induction of anesthesia, which may not reflect a true resting state and may limit its value as an individualized intraoperative target. Third, we used a fixed MAP threshold of 65 mmHg to define hypotension, although emerging data support the concept of individualized blood pressure targets based on preoperative measurements [29]. The current HPI algorithm uses this fixed threshold for all patients, which may reduce its predictive value in certain populations. Future developments should consider customizable MAP targets to better reflect patient-specific needs. Furthermore, the optimal timing and method for determining both baseline cardiac index and individual MAP targets remain unclear. Further research is needed to establish evidence-based protocols for personalizing hemodynamic management in the perioperative setting. Finally, our findings may not be generalizable to other surgical populations or to settings with different anesthetic techniques and perioperative care pathways.

## Conclusion

This study adds important insight from a special patient population and demonstrates that only an HPI-guided algorithm and not a “classical GDT protocol” compared to a standard management protocol can significantly reduce IOH in patients undergoing major maxillofacial and otolaryngological surgery. While no significant differences in secondary outcomes were observed, the findings underscore the potential of individualized, algorithm-driven approaches to improve intraoperative hemodynamic stability. However, given the ongoing debate surrounding clinical relevance, further research is needed to confirm its advantages over standard MAP-based monitoring and to assess its impact on postoperative outcomes in larger, more diverse patient populations.

## Data Availability

The datasets used and/or analysed during the current study are available from the corresponding author on reasonable request.
